# Age and sex-related upper body performance differences in competitive young tennis players

**DOI:** 10.1371/journal.pone.0221761

**Published:** 2019-09-03

**Authors:** Jaime Fernandez-Fernandez, Fabio Yuzo Nakamura, Victor Moreno-Perez, Alejandro Lopez-Valenciano, Juan Del Coso, Cesar Gallo-Salazar, David Barbado, Iñaki Ruiz-Perez, David Sanz-Rivas

**Affiliations:** 1 Department of Physical Activity and Sports Sciences, Universidad de León, León, Spain; 2 Tennis Performance Research Group, Madrid, Spain; 3 Associate Graduate Program in Physical Education UPE/UFPB, João Pessoa, Paraíba, Brazil; 4 Department of Sport Science, Sport Research Centre, Miguel Hernandez University of Elche, Alicante, Spain; 5 Exercise Physiology Laboratory, Camilo José Cela University, Madrid, Spain; 6 Spanish Tennis Federation, Madrid, Spain; University of Brasilia, BRAZIL

## Abstract

**Objective:**

The aims of this study were to analyze the shoulder functional profile of young male and female tennis players and to establish the relationship among physical variables and serve speed.

**Methods:**

A total of 128 Spanish tennis players (Under-13 (n = 32/32 males/females) and Under-15 (n = 36/28 males/females), were tested during National training camps. Tests included passive shoulder range of motion (ROM) for both internal (IR) and external rotation (ER) and isometric strength (i.e., IR and ER) of the dominant/non-dominant shoulders, medicine ball throws (MBT), and serve speed. Age and sex pairwise comparisons were carried using the Hedges’ g index (*d*_*g*_).

**Results:**

Results showed age and sex effects on serve speed and all MBT, with males showing greater changes (1.51≤*d*_*g*_≤1.98) with age than females (0.92≤*d*_*g*_≤1.35; *p*<0.05). U15 males showed higher (*p*<0.05) absolute shoulder IR and ER strength than U13, with only significant differences between males and females in the U15. Regarding ROM, U15 males showed a decreased IR ROM compared to U13 (*d*_*g*_ = -0.84; *p*<0.05) and higher significant IR bilateral deficit (*d*_*g*_ = 0.51; *p*<0.05). The distances obtained in the different MBT were the variables more correlated to serve speed.

**Conclusion:**

The present results suggest that shoulder strength, medicine ball throws and serve speed increased along with age in young elite tennis players of both sexes. However, a decreased range of motion and bilateral deficit for glenohumeral internal rotation is evident in male under-15 tennis players. Muscle strength, power and shoulder range of motion are key factors for serve speed in young tennis players.

## Introduction

It is well known that tennis is an early initiation sport which leads players to spend a lot of training time mastering their individual sport-specific skills since early ages [1]. In this regard, it has been reported that technical and tactical training in young tennis players often exceed 15 to 20 hours per week [2]. In the last few years, several studies suggested that sport specialization and intensive training during adolescent growth stages represent important risk factors for overuse injury in young players, which can reduce long-term performance and hinder the development of a professional career [3–5].

As a result of the demands induced by the continuous practice and play, tennis players are susceptible to a range of injuries including chronic overuse conditions and acute traumatic injuries [6]. Players are required to execute hundreds of strokes per training/match, with powerful shots including serves and groundstrokes [7,8]. The serve, in particular, is the most important shot in competitive tennis, allowing the player to win the point directly through an ace or dominate the rally since the beginning [9]. In terms of performance, although previous research is still scarce, results reported a positive relationship between serve velocity and shoulder strength (i.e., internal (IR) and external rotation (ER)) [10], shoulder range of motion (ROM) (i.e., IR of the dominant shoulder) [11]. In addition, overhead medicine ball throw and serve velocity are the most correlated predictors of overall tennis performance [12].

The serve is the most demanding stroke in tennis, with supra-physiologic forces through the shoulder and elbow [6,13]. Thus, a considerable proportion of injuries in tennis players are located in the dominant shoulder, with an incidence of 8.2 injuries per 1000 playing hours in tennis matches, accounting for 15.9% of all overuse injuries in elite junior tennis players [6,14,15]. These injuries are mainly caused by the conjunction of unilateral and repetitive tennis strokes and biomechanical and training load errors, which lead to alterations in the shoulder ROM [16,17], and to imbalances in the muscle strength [18,19].

The glenohumeral internal rotation deficit (GIRD) (i.e., loss in dominant shoulder IR that is greater than 18–20°, with a corresponding loss of total arc of motion (TAM) greater than 5° when compared with the non-dominant shoulder) [20], external/internal (ER/IR) rotation strength ratios (i.e., <60–85%) [21], or an external rotation deficiency (ERD) (loss in dominant shoulder ER that is greater than 5°, when compared with the non-dominant shoulder [22]), have been highlighted as one of the main risk factors in overhead athletes. Moreover, most of these changes have been related to the duration of tennis practice and player’s age [23,24]. To the best of our knowledge, only few previous studies have been reported the shoulder functional profile (i.e., ROM and strength) of young tennis players (7 to >16 years) [19,25]. In general, IR ROM decreases with age, with a parallel decrease in the total arc of motion (TAM) in the dominant side when compared to the non-dominant side. Moreover, absolute/normalized shoulder strength values were higher in the dominant side compared to the non-dominant side, and values increased with age, although studies showed some discrepancies, especially related to the biological age or when strength data are normalized [19,25].

Therefore, the purpose of this study was twofold: first, to analyze the shoulder functional profile (rotation ROM and strength) of young (under (U)-13 and U-15) male and female tennis players and, second, to establish the relationship between these physical variables (i.e., shoulder profile, medicine ball throws) and serve velocity.

## Method

### Experimental approach to the problem

The current investigation is an observational and descriptive analysis to determine age and sex differences in the shoulder functional profile, medicine ball throws) and serve velocity. Testing protocols were conducted over a 4-week period beginning at the end of September 2017. Test sessions were undertaken between 10:00 and 15:00 hours, and the players were tested at their respective federation base. To ensure standardization of test administration across the entire study period, all tests were performed in the same order, using the same testing devices, measurement protocols and operators. The testing took place in 2 different locations in each federation base: a physiotherapy room and an outdoor synthetic court (Rebound Ace surface; temperature, 22.3–24.4°C; relative humidity, 54.4–61.0%; Kestrel 4000 Pocket Weather Tracker, Nielsen Kellerman, Boothwyn, PA, USA). Every player followed the same testing protocol (i.e., shoulder ROM, shoulder strength, medicine ball throws and serve speed) separated by lapses of 10 min between each stage. To reduce the interference of uncontrolled variables, participants stayed at the same residence within the training facility to control meals and resting times. Participants were encouraged to withdraw all sources of caffeine for 24 h before testing and to have their habitual breakfast at least 3 h before the onset of the measurements. Testing began after a 15-min standardized warm-up, which consisted of jump rope activation, general dynamic mobility, multi-directional acceleration runs, jumps of progressive intensity, and shoulder exercises with elastic tubbing.

### Participants

One hundred and twenty-eight junior tennis players took part in this study (Table 1). For the purposes of the present study, the players were grouped into two age groups: U13 years (32 males and 32 females) and U15 (36 males and 28 females). Participants comprised the most talented players in each region and were selected by the regional federations coaching staffs based on technical or tactical abilities and competitive performance. All players participated in an average of 14 ± 3.1 hours of combined tennis and physical training per week, and had a training background of 6.6 ± 3.2 years. None of the players reported history of any orthopedic problems of any body part during the previous 12 months.

**Table 1:** Descriptive variables of male and female junior tennis athletes according to their age.

		U13		U15	
		n	mean (SD)	n	mean (SD)
**Age (years)**	Male	32	12.6 (0.2)	36	14.6 (0.3)
	Female	32	12.6 (0.3)	28	14.6 (0.3)
**Height (m)**	Male	32	154.9 (7.0)	36	169.0 (5.7)
	Female	32	159.8 (7.0)	28	166.3 (5.7)
**Mass (kg)**	Male	32	43.5 (6.8)	36	58.4 (7.3)
	Female	32	49.1 (7.3)	28	56.8 (5.4)
**APHV (years)**	Male	32	15.0 (0.5)	36	15.4 (0.5)
	Female	32	12.4 (0.4)	28	13.0 (0.4)
**PHV (years)**	Male	32	-2.41 (0.5)	36	-0.74 (0.6)
	Female	32	0.23 (0.5)	28	1.67 (0.4)

U13 = under 13 years old; U15 = under 15 year old; APHV = age at peak height velocity; PHV = peak height velocity; m = meters; kg = kilograms.

Before taking part in the study, participants and their parents/guardians were fully informed about the protocol and provided their written informed consent. The Spanish Tennis Federation Ethics committee approved the procedures in accordance with the latest version of the Declaration of Helsinki.

#### Maturity status

Body height was measured using a fixed stadiometer (60.1 cm; Holtain Ltd., Crosswell, United Kingdom), sitting height using a purpose-built table (60.1 cm; Holtain Ltd.), and body mass using a digital balance (60.1 kg; ADE Electronic Column Scales, Hamburg, Germany). Pubertal timing was estimated according to the biological maturation of each individual using the predictive equation described by Mirwald et al. [26]. The age of peak linear growth (age at peak height velocity) is an indicator of somatic maturity representing the time of maximum growth in stature during adolescence [26]. Biologic age of maturity (in years) was calculated by subtracting the chronological age at the time of measurement from the chronological peak velocity age [27]. Thus, a maturity age of -1.0 indicates that the player was measured 1 year before this peak velocity, a maturity of 0 indicates that the player was measured at the time of this peak velocity, and a maturity age of +1.0 indicates that the participant was measured 1 year after this peak height velocity [26].

#### Shoulder range of motion (ROM)

The passive glenohumeral rotation was assessed following the methodology previously described [28] using a manual inclinometer (ISOMED inclinometer, Portland, Oregon). Each participant lay supine on a bench, with the shoulder in 90° of abduction and the elbow flexed to 90° (forearm perpendicular to the bench). From this starting position, an examiner held the participant’s proximal shoulder region (i.e. clavicle and scapula) against the bench to stabilize the scapula while another examiner rotated the humerus in the glenohumeral joint to produce maximum passive ER and IR [17]. Two attempts at both IR and ER, as well as for both, dominant and non-dominant sides, were performed. Values (°) for both repetitions were averaged, and then used to calculate both TAM, and the bilateral difference in IR (side-to-side asymmetry = dominant—non-dominant; [17]).

#### Shoulder strength test

Isometric internal and external shoulder rotation strength of the dominant and non-dominant limb were assessed with a portable handheld dynamometer (Nicholas Manual Muscle Tester, Lafayette Indiana Instruments). Participants were in a supine lying position on a plinth with the shoulder in 90° of abduction and the elbow flexed to 90° and following the methodology described previously [21]. Strong verbal encouragement was given during every repetition to promote a maximal effort. The average of two maximal trials (5 s) was used for the subsequent statistical analyses. There was a 30-s rest period between trials. A side-to-side difference higher than 10% was defined as bilateral asymmetry. Moreover, shoulder rotational strength values normalized to bodyweight (N/kg) were also calculated [19].

#### Serve speed

The serve speed was measured by a radar gun (model SR3600, Homosassa, FL, USA; range 80 to 232 km/h) and using new tennis balls (Babolat Team). Before each experimental session, the radar gun was calibrated in accordance with the manufacturer’s specifications. According to previous research [29], the radar was positioned on the center of the baseline, 3 m behind the server, aligned with the approximate height of ball contact (~ 2.2 m) and pointing down the center of the court. Each participant carried out 3 sets of 10 maximal flat serves (i.e., the use of slice was not allowed) to the advantage court with a 30 s rest between each of them and approximately 10 s between each serve. To be accepted, serves had to fall into the service box within 1 m of the center service line. Direct feedback of velocities was provided to encourage maximal effort. Before testing, a specific 5 min serve warm-up was allowed to the participants including upper body mobility and 2 sets of 8 first and second serves. Finally, the average speed of the 8 best trials was used for further analysis.

#### Medicine ball throws (MBT): Overhead, forehand, and backhand

For the overhead MBT (MBO), the players stood on a line with their feet side-by-side and slightly apart, facing the direction to which the ball was to be thrown, and holding a 2-kg medicine ball. The ball was brought back behind the head and then thrown vigorously forward as far as possible without the player crossing the line. Additionally, players performed a forehand (MBF) and backhand MBT (MBB) according to previous methods [12]. Players stood sideways to the starting line and simulated a forehand-backhand stroke tossing the ball as far as possible without crossing the line. For all MBT, the distance from the line to the point where the ball landed was measured, and the best performance between 2 efforts was recorded to the nearest 5 cm. There was a 45-s rest period between trials.

### Statistical analyses

Descriptive statistics (mean and standard deviation) were calculated for each of the variables. Data normality was examined using the Kolmogorov-Smirnov test with the Lilliefors’ correction. In order to investigate the differences caused by the maturation and tennis training, two-way independent-measures analyses of variance (ANOVAs) were performed, being age group category (2 levels: U13 and U15) and sex (2 levels: males and females) the between-subject factors. The level of significance chosen was p < 0.05. Additionally, pairwise comparisons were carried using the Hedges’ g index (dg) and its confidence interval at 95% as effect size estimator [30]. This index is based on Cohen’s d index, but it provides an effect size estimation reducing the bias caused by small samples. A comparison was considered statistically significant when the effect size confidence interval did not cross the zero value. Additionally, the practical significance of effect sizes was categorized as trivial (dg < 0.2), small (0.2 ≤ dg < 0.5), moderate (0.5 ≤ dg < 0.8) and large (dg ≥ 0.8). Pearson correlations were used to determine the relationship between performance parameters and serve speed, controlled by age. Correlations were classified as trivial (0–0.1), small (0.1–0.3), moderate (0.3–0.5), large (0.5–0.7), very large (0.7–0.9), nearly perfect (0.9), and perfect (1.0). ANOVA and correlational analysis were performed with the Statistical Package for Social Sciences (version 22.0, SPSS Inc., Chicago, IL, USA). Effect sizes (ES) were calculated through an “ad hoc” excel spreadsheet (21).

In order to identify a group of factors that were independently associated with serve speed, all potential factors that showed significant associations with this parameter and met the assumptions of normality, linearity, homoscedasticity, and non-presence of multicollinearity were entered into a stepwise multivariate linear least square regression with backward elimination (p ≤ 0.1) [31]. The strength of the predictive ability of identified factors was determined with unstandardized regression coefficients (β), while the predictive power of each final model was given by calculation of the percentage of explained variance (R^2^). Both, correlational and multiple regression analysis were performed for each males and females age-group, independently. Potential confounding variables (age, mass and height) were included in the regression model.

## Results

### Serve speed and medicine ball throw

Overall, the ANOVAs revealed effects of *age* (55.766 ≤ F ≤ 72.432; *p* < 0.05) and *sex* (4.275 ≤ F ≤ 26.041; *p* < 0.05) on serve speed and all the three medicine ball throws (MBO, MBF and MBB) (Table 2). There was also an interaction effect (*Age* × *Sex*; 5.107 ≤ F ≤ 14.221; *p* < 0.05) for all these parameters, highlighting that males showed greater changes (1.51 ≤ *d*_*g*_ ≤ 1.98) with age than females (0.92 ≤ *d*_*g*_ ≤ 1.35). Thus, U15 males, but not U13, showed higher serve speed and medicine ball throw distance than U15 and U13 females.

**Table 2:** Differences between male and female tennis players under 13 and 15 years old on speed tennis serve and medicine ball throw distance.

		Males	Females	*Sex*	*Effect size*
**Serve speed (km/h)**	**U13**	126.42 (10.16)	121.95 (10.59)	26.041 (<0.001)	0.43 (-0.07; 0.92)
	**U15**	149.10 (12.27)	132.40 (11.91)		1.36 (0.81; 1.91)
	*Age*	63.751 (<0.001)		*Interaction*	
	*Effect size*	1.98 (1.40; 2.56)	0.92 (0.38; 1.45)	8.702 (0.004)	
**MBO (m)**	**U13**	5.53 (1.18)	5.67 (0.70)	6.581 (0.012)	-0.14 (-0.63; 0.35)
	**U15**	7.79 (1.35)	6.65 (0.74)		1.00 (0.48; 1.52)
	*Age*	69.194 (<0.001)		*Interaction*	
	*Effect size*	1.76 (1.19; 2.32)	1.35 (0.78; 1.91)	10.785 (0.001)	
**MBF (m)**	**U13**	7.25 (1.17)	7.12 (0.95)	18.229 (<0.001)	0.12 (-0.38; 0.61)
	**U15**	10.31 (1.90)	8.30 (0.99)		1.26 (0.72; 1.80)
	*Age*	72.432 (<0.001)		*Interaction*	
	*Effect size*	1.89 (1.32; 2.47)	1.20 (0.65; 1.75)	14.221 (<0.001)	
**MBB (m)**	**U13**	6.80 (1.20)	6.85 (0.92)	4.275 (0.041)	-0.04 (-0.53; 0.45)
	**U15**	9.25 (1.88)	8.16 (1.13)		0.67 (0.17; 1.18)
	*Age*	55.766 (<0.001)		*Interaction*	
	*Effect size*	1.51 (0.97; 2.05)	1.26 (0.71; 1.82)	5.107 (0.026)	

Two way independent measures ANOVAs being sex and age the between subject factors. ANOVA main effects (sex; Age) and interactions (sex × age) are presented as F score (p). Descriptive data are presented as mean (SD). Effect sizes were calculated using Hedges’ g index and they are presented as mean (90% confidence interval). MBO: overhead medicine-ball throw; MBF: medicine-ball throw, forehand side; MBB: medicine-ball throw, backhand side.

### Shoulder strength

As shown in Table 3, U15 showed higher absolute shoulder IR and ER strength than U13 (42.039 ≤ F ≤ 55.773; *p* < 0.05). However, differences between males and females were only significant in the U15 (*Age* × *Sex*; 9.507 ≤ F ≤ 14.023; *p* < 0.05). Unlike absolute strength values, males showed higher normalized shoulder strength than females in both age groups (21.895 ≤ F ≤ 29.602; *p* < 0.05). Nevertheless, no significant differences between age groups were observed for normalized shoulder strength. Finally, ER/IR strength ratio and strength bilateral deficit did not show significant changes with *age* and *sex*.

**Table 3:** Differences between male and female tennis players under 13 and 15 years old on shoulder internal and external rotation strength.

		Males	Females	*Sex*	*Effect size*
*Dominant arm*					
**APF**_**IR**_ **(N)**	**U13**	114.8 (21.9)	111.1 (15.2)	16.051 (<0.001)	0.19 (-0.30; 0.68)
	**U15**	156.1 (32.8)	125.9 (19.1)		1.08 (0.55; 1.60)
	*Age*	44.163 (<0.001)		*Interaction*	
	*Effect size*	1.45 (0.91; 1.98)	0.86 (0.32; 1.39)	9.810 (0.002)	
**NPF**_**IR**_ **(N/kg)**	**U13**	2.70 (0.56)	2.29 (0.32)	28.963 (<0.001)	0.89 (0.37; 1.40)
	**U15**	2.68 (0.47)	2.23 (0.37)		1.02 (0.49; 1.54)
	*Age*	0.269 (0.605)		*Interaction*	
	*Effect size*	-0.05 (-0.52; 0.43)	-0.16 (-0.67; 0.34)	0.043 (0.836)	
**APF**_**ER**_ **(N)**	**U13**	89.4 (21.0)	86.5 (15.6)	14.629 (<0.001)	0.15 (-0.34; 0.64)
	**U15**	126.0 (28.9)	99.5 (15.9)		1.08 (0.55; 1.61)
	*Age*	42.039 (<0.001)		*Interaction*	
	*Effect size*	1.42 (0.89; 1.95)	0.82 (0.29; 1.34)	9.507 (0.003)	
**NPF**_**ER**_ **(N/kg)**	**U13**	2.08 (0.44)	1.79 (0.34)	25.750 (<0.001)	0.73 (0.23; 1.24)
	**U15**	2.15 (0.43)	1.76 (0.26)		1.06 (0.53; 1.59)
	*Age*	0.104 (0.747)		*Interaction*	
	*Effect size*	0.16 (-0.31; 0.64)	-0.09 (-0.60; 0.42)	0.550 (0.460)	
*Non dominant arm*					
**APF**_**IR**_ **(N)**	**U13**	93.2 (13.3)	91.9 (17.6)	16.850 (<0.001)	0.08 (-0.41; 0.57)
	**U15**	133.5 (27.8)	104.8 (19.4)		1.16 (0.63; 1.69)
	*Age*	52.672 (<0.001)		*Interaction*	
	*Effect size*	1.79 (1.23; 2.36)	0.69 (0.17; 1.21)	14.023 (<0.001)	
**NPF**_**IR**_ **(N/kg)**	**U13**	2.20 (0.40)	1.90 (0.38)	29.602 (<0.001)	0.76 (0.25; 1.27)
	**U15**	2.28 (0.37)	1.86 (0.34)		1.18 (0.64; 1.71)
	*Age*	0.109 (0.742)		*Interaction*	
	*Effect size*	0.22 (-0.26; 0.70)	-0.11 (-0.62; 0.40)	0.885 (0.349)	
**APF**_**ER**_ **(N)**	**U13**	84.0 (15.8)	77.4 (12.1)	10.235 (0.002)	0.46 (-0.04; 0.95)
	**U15**	112.4 (24.9)	98.0 (17.1)		0.65 (0.15; 1.16)
	*Age*	55.773 (<0.001)		*Interaction*	
	*Effect size*	1.33 (0.80; 1.86)	1.38 (0.82; 1.95)	1.463 (0.229)	
**NPF**_**ER**_ **(N/kg)**	**U13**	1.95 (0.29)	1.61 (0.31)	21.895 (<0.001)	1.14 (0.62; 1.67)
	**U15**	1.92 (0.36)	1.73 (0.31)		0.54 (0.04; 1.05)
	*Age*	0.703 (0.404)		*Interaction*	
	*Effect size*	-0.10 (-0.57; 0.38)	0.41 (-0.10; 0.92)	1.984 (0.162)	
*External/internal rotation strength ratio*					
**Dominant arm (Unitless)**	**U13**	0.78 (0.27)	0.78 (0.13)	0.031 (0.861)	-0.03 (-0.52; 0.46)
	**U15**	0.80 (0.24)	0.81 (0.18)		-0.04 (-0.54; 0.45)
	*Age*	0.467 (0.495)		*Interaction*	
	*Effect size*	0.08 (-0.40; 0.56)	0.15 (-0.35; 0.66)	0.131 (0.718)	
**Non-dominat arm (Unitless)**	**U13**	0.93 (0.24)	0.86 (0.14)	0.952 (0.331)	0.37 (-0.13; 0.86)
	**U15**	0.83 (0.22)	0.96 (0.22)		-0.56 (-1.06; -0.06)
	*Age*	0.139 (0.710)		*Interaction*	
	*Effect size*	-0.41 (-0.89; 0.07)	0.55 (0.03; 1.07)	4.459 (0.037)	
*Bilateral strength differences*					
**Internal rotation (%)**	**U13**	23.32 (17.26)	22.96 (17.66)	0.306 (0.581)	-0.32 (-0.82; 0.17)
	**U15**	18.28 (17.85)	22.14 (18.00)		0.53 (0.03; 1.03)
	*Age*	0.862 (0.355)		*Interaction*	
	*Effect size*	-0.28 (-0.76; 0.19)	-0.05 (-0.55; 0.46)	.446 (0.506)	
**External rotation (%)**	**U13**	7.03 (17.64)	12.66 (16.81)	0.592 (0.443)	0.37 (-0.13; 0.86)
	**U15**	13.57 (23.86)	2.79 (14.00)		-0.56 (-1.06; -0.06)
	*Age*	0.247 (0.620)		*Interaction*	
	*Effect size*	0.31 (-0.17; 0.78)	-0.63 (-1.14; -0.11)	6.008 (0.016)	

*Two way independent measures ANOVAs being sex and Age the between subject factors*. *ANOVA main effect (sex*, Age) and interaction (sex*Age) are presented as F score (p). *Descriptive data are presented as mean (SD)*. *Effect sizes were calculated using Hedges’ g index and they are presented as mean (90% confidence interval)*. APF_IR_: absolute internal rotation peak force; NPF_IR_: normalized internal rotation peak force; APF_ER_: absolute external rotation peak force; NPF_ER_: normalized external rotation peak force.

### Shoulder ROM

As shown in Table 4, overall, there were no meaningful differences in shoulder IR, ER, ROM and neither in TAM between sex and age groups. However, pairwise comparisons showed that U15 males showed a decreased IR ROM compared to U13 (U13 = 73.0°; U15 = 61.8°; *d*_*g*_ = -0.84; *p* <0.05). In addition, they showed a higher and statistically significant IR bilateral deficit (U13 = 12.0%; U15 = 20.1%; *d*_*g*_ = 0.51; *p* <0.05).

**Table 4:** Differences between males and female tennis players under 13 and 15 years old on shoulder internal and external rotation range of motion.

		Males	Females	*Sex*	*Effect size*
**D-IR (°)**	**U13**	73.0 (12.1)	67.1 (14.1)	0.474 (0.492)	0.44 (-0.05; 0.94)
	**U15**	61.8 (13.8)	71.0 (10.5)		-0.72 (-1.23; -0.21)
	*Age*	2.368 (0.127)		*Interaction*	
	*Effect size*	-0.84 (-1.34; -0.35)	0.31 (-0.20; 0.82)	10.238	(0.002)
**ND-IR (°)**	**U13**	81.35 (11.00)	80.09 (17.62)	0.259 (0.612)	0.08 (-0.41; 0.57)
	**U15**	77.60 (10.75)	81.39 (13.85)		-0.31 (-0.80; 0.19)
	*Age*	0.240 (0.625)		*Interaction*	
	*Effect size*	-0.34 (-0.82; 0.14)	0.08 (-0.43; 0.59)	1.021 (0.314)	
**D-ER (°)**	**U13**	146.6 (18.5)	140.1 (18.2)	0.517 (0.474)	0.35 (-0.14; 0.85)
	**U15**	136.4 (14.6)	138.4 (17.3)		-0.13 (-0.62; 0.37)
	*Age*	3.609 (0.060)		*Interaction*	
	*Effect size*	-0.61 (-1.10; -0.12)	-0.09 (-0.60; 0.42)	1.897 (0.171)	
**ND-ER (°)**	**U13**	140.69 (14.12)	139.97 (11.90)	0.857 (0.357)	0.05 (-0.44; 0.54)
	**U15**	134.49 (15.29)	140.00 (15.00)		-0.36 (-0.86; 0.14)
	*Age*	1.423 (0.235)		*Interaction*	
	*Effect size*	-0.42 (-0.90; 0.07)	0.00 (-0.50; 0.51)	1.452 (0.231)	
**D-TAM (°)**	**U13**	219.58 (26.31)	207.13 (25.53)	0.020 (0.888)	0.47 (-0.02; 0.97)
	**U15**	198.20 (23.44)	209.39 (21.87)		-0.49 (-0.99; 0.02)
	*Age*	1.471 (0.228)		*Interaction*	
	*Effect size*	-0.85 (-1.35; -0.35)	0.09 (-0.41; 0.60)	7.065 (0.009)	
**ND-TAM (°)**	**U13**	222.04 (21.65)	220.06 (24.05)	0.798 (0.374)	0.09 (-0.40; 0.58)
	**U15**	212.09 (19.96)	221.39 (24.06)		-0.42 (-0.92; 0.08)
	*Age*	1.104 (0.296)		*Interaction*	
	*Effect size*	-0.47 (-0.96; 0.01)	0.05 (-0.45; 0.56)	1.890	(0.172)
**IR bilateral** **differences (%)**	**U13**	10.10 (10.84)	14.86 (18.13)	0.487 (0.487)	-0.31 (-0.81; 0.18)
	**U15**	20.08 (15.70)	11.45 (13.91)		0.57 (0.07; 1.07)
	*Age*	1.412 (0.237)		*Interaction*	
	*Effect size*	0.72 (0.23; 1.22)	-0.21 (-0.71; 0.30)	5.852 (0.017)	
**TAM bilateral** **differences (°)**	**U13**	2.46 (15.82)	12.94 (30.56)	1.221 (0.271)	-0.43 (-0.92; 0.07)
	**U15**	13.89 (17.37)	12.00 (16.46)		0.11 (-0.38; 0.60)
	*Age*	1.820	(0.180)	*Interaction*	
	*Effect size*	0.68 (0.19; 1.17)	-0.04 (-0.54; 0.47)	2.529	(0.115)

Two way independent measures ANOVAs being sex and Age the between subject factors.

ANOVA main effect (sex, Age) and interaction (sex*Age) are presented as F score (p). Descriptive data are presented as mean (SD). Effect sizes were calculated using Hedges’ g index and they are presented as mean (90% confidence interval).

D: dominant arm; ND: non-dominant arm; IR: internal rotation; ER: external rotation; TAM: total arc of motion; IR ratio was calculated as the IR range of motion difference between non-dominant and dominant arm, divided by non-dominant IR range of motion and multiplied by 100.

### Correlational analysis

In males (Table 5), medicine-ball throws showed the highest correlations with serve speed (0.418 < *r* < 0.638), being the MBF throw the best predictor (U13: 0.582; U15: 0.638). Regarding shoulder strength, absolute IR and ER strength were significantly correlated with serve speed, for both, U13 (IR: 0.518; ER: 0.472) and U15 group (IR: 0.496; ER: 0.391). Interestingly, in U15 males, the IR ROM was negatively correlated with serve speed (*r* = -0.369). This significant association between serve speed and ROM was not observed in any other group. Regarding anthropometrical measures, body height (0.549 < *r* < 0.594) and body weight (0.600 < *r* < 0.625) were significantly correlated with serve velocity for both, the U15 and U13 groups.

**Table 5:** Correlations between served speed, medicine-ball throws, shoulder rotation strength and range of motion in males under 13 (top-right) and 15 years old (left-bottom).

	**Serve speed**	**MBO**	**MBF**	**MBB**	**APF**_**IR**_	**NPF**_**IR**_	**APF**_**ER**_	**NPF**_**ER**_	**ER/IR ratio**	**BD-PF**_**IR**_	**BD-PF**_**ER**_	**ROM**_**IR**_	**ROM**_**ER**_	**TAM**	**BD-ROM**_**IR**_	**BD-TAM**	**Height**	**Mass**
**Serve speed**		**.418**	**.582**	**.532**	**.518**	.055	**.472**	.134	.065	**.360**	.187	.141	-.106	-.010	**-.446**	-.204	**.549**	**.625**
**MBO**	**.557**		**.734**	**.630**	**.445**	.025	**.390**	.047	.006	.301	.034	-.031	-.213	-.163	.024	.228	**.494**	**.526**
**MBF**	**.638**	**.637**		**.865**	**.353**	-.073	.342	-.013	.065	.143	.161	-.257	-.197	-.256	-.025	.067	**.660**	**.572**
**MB**_**B**_	**.442**	**.592**	**.813**		**.405**	-.036	**.403**	.057	.060	.098	.152	-.187	-.260	-.268	-.162	.064	**.633**	**.535**
**APF**_**IR**_	**.496**	.240	.332	.226		**.720**	.245	.105	**-.541**	**.655**	.163	.191	.152	.195	.058	.118	.132	.217
**NPF**_**IR**_	.150	-.033	-.029	-.148	**.797**		-.130	.191	**-.667**	**.537**	.152	.253	**.501**	**.467**	.108	-.039	**-.394**	**-.470**
**APF**_**ER**_	**.391**	.252	**.627**	**.607**	**.527**	.245		**.783**	**.649**	.049	**.506**	-.274	**-.413**	**-.415**	.113	.127	**.476**	**.471**
**NPF**_**ER**_	.109	.046	**.366**	**.360**	.326	.354	**.868**		**.553**	-.040	**.608**	-.181	-.069	-.131	.206	.061	.023	-.149
**ER/IR ratio**	.028	.089	**.383**	**.449**	**-.374**	**-.538**	**.571**	**.578**		**-.451**	.253	-.365	**-.434**	**-.472**	.021	.022	**.380**	.298
**BD-PF**_**IR**_	-.297	-.299	**-.412**	**-.360**	.314	**.489**	-.064	.018	**-.438**		-.052	.184	.280	.281	-.048	-.120	-.082	.081
**BD-PF**_**ER**_	.116	.117	.218	.100	**.428**	**.473**	**.403**	**.455**	.046	.307		.132	.111	.139	.158	-.035	.062	-.032
**ROM**_**IR**_	**-.369**	-.179	-.259	-.036	.202	.322	.078	.178	-.190	.323	.283		**.463**	**.783**	**-.593**	**-.393**	-.134	-.103
**ROM**_**ER**_	-.186	-.195	**-.442**	**-.347**	.029	.271	-.181	.012	-.204	.311	.108	**.356**		**.914**	-.121	**-.554**	-.367	**-.454**
**TAM**	-.334	-.228	**-.428**	-.237	.137	**.359**	-.067	.112	-.239	**.384**	.234	**.813**	**.834**		-.356	**-.569**	-.319	-.365
**BD-ROM**_**IR**_	.236	-.039	.158	.019	-.070	-.141	.054	-.014	.210	-.067	-.153	**-.795**	-.085	**-.523**		**.602**	-.204	-.146
**BD-TAM**	.307	.207	**.455**	.254	.094	-.039	.208	.103	.220	-.217	-.005	**-.681**	-.247	**-.556**	**.751**		.172	.056
**Height**	**.594**	**.378**	**.487**	**.597**	**.459**	.036	**.562**	.265	.204	-.111	.107	.064	-.063	-.002	-.023	.033		**.783**
**Body Mass**	**.600**	**.449**	**.612**	**.610**	**.506**	-.114	**.533**	.050	.173	-.183	.044	-.128	**-.337**	-.286	.104	.233	**.693**	

Correlations were only performed with the dominant shoulder. Significant correlations are presented in bold.

Serve speed (km/h); MBO: overhead medicine-ball throw; MBF: medicine-ball forehand side; MBB: medicine-ball backhand side;APFIR: absolute internal rotation peak force; NPFIR: normalized internal rotation peak force; APFER: absolute external rotation peak force; NPFER: normalized external rotation peak force; PF_ER/IR_ ratio: shoulder external/internal rotation peak of force ratio (unitless); ROM_IR_: internal rotation range of motion (°); ROM_ER_: external rotation range of motion (°); TAM: shoulder total arc of motion (°); BD-ROM_IR_: Bilateral deficit of internal rotation range of motion (%); BD-TAM: Bilateral deficit of total arc of motion (°).

In females (Table 6), correlational values showed that in the U13 group only MBF (*r* = 0.413) was significantly correlated with serve speed. In the U15, only MBO (*r* = 0.433) was significantly correlated with serve speed. Regarding anthropometrical measures, only body weight of the U15 group (*r* = 0.489) was significantly correlated with serve velocity. Although non-significant, body height for both, U13 and U15, showed moderate correlations (0.319 < *r* < 0.369) with serve velocity.

**Table 6:** Correlations between served speed, medicine-ball throws, shoulder rotation strength and range of motion in females under 13 (top-right) and 15 years old (left-bottom).

	**Serve speed**	**MBO**	**MBF**	**MBB**	**APF**_**IR**_	**NPF**_**IR**_	**APF**_**ER**_	**NPF**_**ER**_	**ER/IR ratio**	**BD-PF**_**IR**_	**BD-PF**_**ER**_	**ROM**_**IR**_	**ROM**_**ER**_	**TAM**	**BD-ROM**_**IR**_	**BD-TAM**	**Height**	**Mass**
**Serve speed**		.202	**.413**	.098	.178	.133	.117	.059	-.039	-.148	-.088	.050	.225	.189	.136	-.006	.319	.066
**MBO**	**.433**		.358	.147	.250	-.065	**.379**	.124	.210	.050	-.038	-.038	.018	-.008	.040	.063	.256	.256
**MBF**	.295	.250		**.718**	**.406**	-.097	.122	-.228	-.188	.124	-.211	-.344	.004	-.187	.129	-.115	**.582**	**.469**
**MB**_**B**_	.307	.159	**.855**		.324	-.131	.007	-.268	-.225	.164	-.035	-.335	-.045	-.217	.073	-.122	.194	**.407**
**APF**_**IR**_	.250	.212	.288	.102		**.472**	**.417**	.034	-.334	.095	-.103	-.117	.016	-.053	.101	-.004	.088	**.435**
**NPF**_**IR**_	-.037	.053	.090	-.071	**.833**		.123	**.503**	-.229	.051	-.230	-.029	.342	.228	.038	-.081	-.336	**-.581**
**APF**_**ER**_	.373	**.396**	.191	.035	.005	-.191		**.717**	**.710**	-.223	**.565**	-.005	.124	.085	.065	-.069	-.159	.219
**NPF**_**ER**_	.053	.260	.037	-.132	-.082	.100	**.774**		**.720**	-.260	**.428**	.081	.349	.293	.014	-.130	**-.464**	**-.513**
**ER/IR ratio**	.097	.129	-.104	-.068	**-.704**	**-.732**	**.683**	**.578**		-.332	**.645**	.115	.108	.141	-.015	-.061	-.251	-.116
**BD-PF**_**IR**_	.265	.254	.179	.107	.249	.245	-.167	-.165	-.300		-.042	-.001	-.129	-.092	-.285	.002	.071	.034
**BD-PF**_**ER**_	.327	.206	-.358	-.311	-.219	-.238	.322	.225	**.381**	.107		.038	.032	.044	-.117	-.122	-.214	.107
**ROM**_**IR**_	-.330	.180	-.110	-.144	.206	.273	-.159	-.049	-.215	.097	-.162		.236	**.721**	**-.620**	-.248	-.026	-.064
**ROM**_**ER**_	.034	-.076	**.469**	**.432**	.114	.171	-.230	-.135	-.258	.214	-.217	.192		**.843**	-.314	**-.713**	-.010	-.298
**TAM**	-.131	.026	.318	.273	.189	.266	-.258	-.130	-.307	.216	-.249	**.630**	**.883**		**-.567**	**-.645**	-.021	-.247
**BD-ROM**_**IR**_	.232	.019	-.018	.044	-.357	-.338	-.019	-.051	.233	.111	**.406**	-.372	.089	-.107		**.722**	-.011	.018
**BD-TAM**	.073	.060	-.179	-.215	**-.394**	-.340	.076	.084	.329	-.069	**.419**	-.098	-.240	-.237	**.765**		-.033	.030
**Height**	.369	.152	**.722**	**.661**	.109	-.143	.345	.068	.145	.177	-.136	-.290	.108	-.054	.073	.041		**.431**
**Body Mass**	**.489**	.239	.280	.273	.135	**-.427**	**.413**	-.251	.205	.027	.114	-.151	-.123	-.169	.011	-.039	**.469**	

Correlations were only performed with the dominant shoulder. Significant correlations are presented in bold.

Serve speed (km/h); MBO: overhead medicine-ball throw; MBF: medicine-ball forehand side; MBB: medicine-ball backhand side;APFIR: absolute internal rotation peak force; NPFIR: normalized internal rotation peak force; APFER: absolute external rotation peak force; NPFER: normalized external rotation peak force; PF_ER/IR_ ratio: shoulder external/internal rotation peak of force ratio (unitless); ROM_IR_: internal rotation range of motion (°); ROM_ER_: external rotation range of motion (°); TAM: shoulder total arc of motion (°); BD-ROM_IR_: Bilateral deficit of internal rotation range of motion (%); BD-TAM: Bilateral deficit of total arc of motion (°).

The prediction model conducted (Table 7) revealed that in U13 males, 71.3% of the serve speed variance was explained with the MBF, bilateral deficit of IR ROM, ER and IR strength. In the U15, 60.2% of the variance was explained by MBF, body height, IR ROM and IR strength. In U13 females, only the MBF explained 17.1% of the serve speed variance, while in the U15 body weight and MBO explained 34.5% of serve performance.

**Table 7:** Backward multivariate linear regression analysis. Significant predictor variables (*p* ≤ 0.10) for the serve speed.

	Explained variance (R^2^)					Regression equation
	Model	1^st^ Variable	2^nd^ Variable	3^rd^ Variable	4^th^ Variable	
*MALES*						
**U13**	*71*.*3%*	MBT_R_	BD-ROM_IR_	APF_ER_	APF_IR_	Y = 70.858 + 3.530*MBF– 0.472* BD-ROM_IR_ + 0.182*APF_ER_ + 0.157*APF_IR_
		31.8%	18.7%	10.5%	10.3%	
**U15**	*60*.*2%*	MBT_R_	Height	ROM_IR_	APF_IR_	Y = 11.726 + 1.685*MBF+ 0.722*Height– 0.346* ROM_IR_ + 0.125*APF_IR_
		40.6%	10.5%	8.5%	8.0%	
*FEMALES*						
**U13**	*17*.*1%*	MBT_R_				Y = 89.891 + 4.488*MBF
		17.1%				
**U15**	*34*.*5%*	Body Mass	MBT_O_			Y = 49.493 + 0.868*Mass + 5.057*MBO
		23.9%	10.6%			

Serve speed (km/h); MBO: overhead medicine-ball throw (m); MBF: medicine-ball throw, forehand side (m); APF_IR_: Internal rotation absolute shoulder peak force (N); APF_ER_: External rotation absolute shoulder peak force (N); ROM_IR_: shoulder internal rotation range of motion (°); BD-ROM_IR_: Bilateral deficit of shoulder internal rotation range of motion (%).

## Discussion

The aims of this study were first, to analyze the shoulder functional profile (rotation ROM and strength) of young (U-13 and U-15) competitive male and female tennis players and, second, to establish the relationship between physical variables (i.e., shoulder functional profile, medicine ball throws) and serve velocity. Main results showed that, in terms of absolute shoulder strength, U15 males were stronger than U13 peers, but males were only significantly stronger than females in the U15 group. When strength values were expressed relative to the body weight, males were stronger than females in both groups (U15 and U13), with no differences between age groups. Regarding shoulder ROM, results highlighted a decreased IR ROM in U15 males compared to the U13 peers, together with higher and statistically significant IR bilateral deficit. Moreover, although non-significant, there is a decreased TAM of the dominant side in U15 males compared to the U13 males. Analyzing physical performance, U15 males showed higher serve speeds and achieved more distance in the medicine ball throws than U13 males and both, U13 and U15 females. Correlations between physical parameters and serve speed highlighted the medicine ball throws, specially the MBF, as the variables more correlated to serve performance.

Upper body performance (i.e., muscular performance and stroke efficiency) seems to be determinant in tennis, since the serve, forehand and backhand rally balls accounted for 70–80% of the external hitting load during elite competition [32,33]. As shown in previous research [19,25], the absolute isometric shoulder rotation strength of adolescent tennis players is increased along with chronologic age in both sides, with side to side differences existing since early ages. Present results showed that U15s were stronger than U13s in both, males and females, although no differences were found between males and females of the U13 group. However, when the data were normalized to body weight, males showed higher strength values than females in both age groups, although no differences were found when comparing age groups in both, males and females. Results are difficult to compare with previous research [19,25], since the sample of tennis players monitored presented different skill levels and ages or maturation levels. In this regard, Cools et al. [19] reported that normalized strength of the shoulder-complex muscles, except for the IRs, remained similar through adolescence in a group of elite tennis players of 12 to 18 years, while Gillet et al. [25] showed that all normalized strength of the shoulder-complex muscles remained similar across biological age groups analyzed (i.e., elite male players, 7 to 13 years). Since muscular strength increased progressively with both mass and height [34], present results can be considered a normal and expected adaptation.

Intensive tennis practice and competition lead to an unbalanced shoulder function profile, with higher IR strength compared to the ER on the dominant side. In this regard, cut-off values reported in previous research were <60% to 85% (34), while present results showed “healthy” average IR/ER ratios between 78% to 96%. However, analyzing individual ratios, values ranged from 50% (U13 females) to 62.5% (U13 males), which are below cut off references for shoulder injury risk and indicates that some of these young tennis players might have moderate risk of shoulder injury. Comparing sides, players already presented a stronger dominant than non-dominant upper limb, with differences ranging from 15 up to 20% in the IR and ER strength, which is in agreement with previous studies [19,25,28]. Asymmetry levels found are likely due to the high demands imposed on the dominant side, especially on the IR strength, during tennis play since early ages, which can increase the tensile stress on the posterior rotator cuff and scapular stabilizers [28], and could develop a strength imbalance between the ER and IR over time. Thus, it seems that an individual approach should be followed in terms of shoulder profiling, as some players participating in the study already seem to be prone to suffer a shoulder injury in the future.

Extensive research has shown that excessive or limited shoulder ROM may lead to shoulder injuries, such as instability and impingement, in overhead athletes [18,35–37]. The current results showed reductions in IR ROM in the dominant shoulder compared to the non-dominant side, for both sexes and age groups, which are in line with previous results obtained in tennis players [17,19]. This IR ROM reduction is likely caused by a sport-specific adaptation, as discussed in recent research [18]. Moreover, and also in line with recent research [25], U15 males showed significantly less IR ROM as well as higher IR deficit than U13, highlighting a progressive limitation as age increases. Research has identified IR limitations and injury risk when there is a loss of rotation greater than 18° to 20°, with a corresponding loss of TAM greater than 5° when compared bilaterally [18]. Present results showed that IR bilateral differences ranged from 8° to 16° in both sex and age groups, which could be considered “normal”, from a pathological point of view [35]. However, caution should be taken, as some individual values in the current study can be considered dangerous, with bilateral differences exceeding more than 20° in some cases. Regarding bilateral differences in the TAM, values ranged from 2.5° (boys U13) to 13.9° (boys U15), with the latter being already considered as a risk for a shoulder injury in the future. Based on these results, it appears necessary to introduce prevention measures in order to balance these shoulder deficits, even at these early age stages. Although there is not enough evidence to support that a stretching program reduces the incidence of recurrent shoulder injury [18], the use of active, passive or manual therapy forms of stretching, is recommended to improve posterior shoulder tightness and GIRD in the short-term for asymptomatic young overhead athletes.

The results regarding correlations between physical qualities and performance (Tables 5 and 6) revealed interesting associations. In males, results showed that MBTs are strong predictors (r = 0.418–0.638) of serve velocity, together with the absolute IR and ER strength (r = 0.391–0.518) for both age groups. In addition, body height (r = 0.549–0.594) and body mass (r = 0.600–0.625) were also positively correlated to serve velocity. In contrast to males, only MBF (r = 0.413) in the U13, and MBO (r = 0.433), in the U15, together with body weight (r = 0.489) in the U15, were significantly correlated with serve velocity in females. Moreover, although non-significant, body height showed moderate correlations (0.319 < r < 0.369) with serve velocity in both age groups. These results are in agreement with previous findings obtained in male and female youth players pooled together [10,12,30], highlighting the determining role of upper body strength-power on service performance, with a lower influence of anthropometric variables. Hence, despite the proposed sequential transfer of power from the lower-body to the core structure and upper-body extremities (i.e., kinetic chain), it is apparent that the strength and power in the upper limb in relation to the movement initiation segment are key factors of the final velocity in the racket head during the tennis serve [7].

In addition to these fitness-related factors, anthropometric characteristics (i.e., body height and weight) were correlated to serve velocity in both age and sex groups. Higher body heights are associated to higher impact points during the serve [7,38], thus, increasing the probability of having a more successful serve [38]. From a biomechanical point of view, body mass is also related to torque production and thus, having a positive contribution to the serve velocity [30,39].

The prediction model conducted in the present study showed interesting results, with the MBF, bilateral deficit of IR ROM, ER and IR strength explaining 71.3% of the serve speed variance in the U13 males, while MBF, body height, IR ROM and IR strength explained 60.2% of the variance in the U15. In U13 females, only the MBF explained 17.1% of the serve speed variance, while in the U15 body weight and MBO explained 34.5% of serve performance. Although it is difficult to compare results, due to the different populations (i.e., age range, number of players analyzed) and testing procedures, a recent study [30], found similar results in a wider range of tennis players (i.e., 1019 males and females ranging from U12 to U18). Results of this study showed that MBTs, handgrip strength, arm span, body height and body mass were correlated with serve speed, and that a combination of different factors (i.e., MBTs, grip strength, arm span, and body mass) explained 41–66% and 19–45% of the variance in serve speed of boys and girls, respectively. From all the upper body measures conducted here, MBTs (i.e., MBF and MBO) explained 10 to 40% of the serve variance for both, males and female players, in line with previous research [12,30]. Thus, it seems that upper-body power and the ability to transfer power from the lower to the upper body (i.e., coordination), including rotational movements [40], are especially important for tennis performance [12].

A perfect prediction model for serve performance in tennis seems to be very difficult to obtain since that stroke is the most complex in the sport [13], with individual technical skills, as well as coordination being also important factors in producing high ball speeds [41]. From or results we can highlight that that differences between males and females become already evident since early ages, with serve performance relying more on the physical qualities (i.e., MBT, shoulder strength) in males, while in females, only the MBT and body mass seemed to be contributors to serve speed. Results are partially in agreement with previous studies, which found that body mass was a major contributor for serve speeds in females, especially in U14 and U18 players [30,42]. This could be related to the increase in body mass when female athletes reach the adolescence [43], which, on the other hand, is also related to an increase in the absolute/relative fat mass, superior strength and power levels, but also poor endurance, speed/agility levels [44]. In this regard, differences in the performance profile of elite players are well reported, with males showing faster serve speeds, and producing more service points than female players [45]. Moreover, males’ game is characterized by higher mean movement speeds than women’s match play which, together with the different match formats at the high level (i.e., 3 vs 5 sets), highlight the need for coaches and physical trainers to individualize strength and conditioning programs in accordance with the physical profile of players (i.e., males to be conditioned for faster-paced tennis; [45]).

Interestingly, the present model showed that, in the U13 male group, bilateral IR ROM deficit explained 18.7% of the serve variance, while in the U15, IR ROM explained 8.5% of that variance. We are not aware of similar studies and thus, comparisons are difficult. It seems that, as previously reported [19], the IR bilateral deficit is a sport-specific adaptation, and caution should be taken when deficits exceed the recommended values [35]. Moreover, it seems that an optimal IR ROM levels are required to achieve optimal serve speeds, and the use of stretching programs, as previously mentioned, are recommended, not only to improve posterior shoulder tightness and GIRD, but also to maintain optimal performance levels [46].

Some limitations can be found in the present study. Some of the unexplained variance of serve speed could be related to lower body performance (e.g., mechanical power in the triple extension during squat or jump squat) or core-strength that were not tested. In addition, measuring maximal serve speed does not account for the complex techniques involved in this tennis-specific skill, which can be used to be effective even while performing submaximal speed serves (e.g., second serve with slice and sidespin). In other words, future studies need to address the technical variations of serve without analyzing solely its speed.

## Conclusions

Since the serve is the most important stroke in tennis, it seems important to determine the physical qualities that define its performance (i.e., speed) and/or help to prevent a future overuse injury, particularly in the shoulder. The present results showed that shoulder strength, medicine ball throws distance and serve speed are increased in the transition from U13 to U15 age categories in elite tennis players of both sexes. However, male U15 players showed a decreased glenohumeral IR compared to U13, accompanied with higher IR bilateral deficit. In the power-based variables, males showed greater differences between age categories than females, leading to significant differences between sexes in the U15 category. A greater proportion of variance in serve speed could be explained by a combination of medicine ball throwing performance, ROM and shoulder strength in males (60.2 to 71.3%), compared to females (17.1 to 34.5%). This suggests that muscle strength, power and range of motion are key factors for serve speed but other factors can also aid to explain tennis serve performance, particularly in females.

As an application of the main results obtained in this investigation, the implementation of training programs aimed at increasing upper body power would be highly recommended at this stage of development (i.e., pre and post-PHV), with previous research showing that the inclusion of combined programs (i.e., MBT, elastic bands and core strength) can be helpful for increasing performance levels at these ages [46–48]. Moreover, from an injury prevention perspective, it appears necessary to maintain and/or improve “normal” shoulder ROMs when needed, since early ages [35].
